# DMAIC methodology for achieving public satisfaction with health departments in various districts of Punjab and optimizing CT scan patient load in urban city hospitals

**DOI:** 10.3934/publichealth.2022030

**Published:** 2022-05-10

**Authors:** Muhammad Mutasim Billah Tufail, Asad Shamim, Asghar Ali, Muhammad Ibrahim, Danial Mehdi, Waseem Nawaz

**Affiliations:** Department of Management Studies, Bahria University, Karachi Campus

**Keywords:** six-sigma, DMAIC, healthcare management, CT scan, house of quality (HOQ)

## Abstract

The health care delivery system in Pakistan is in a process of rapid growth, and it consists of public and private sectors. The provincial government is preliminarily responsible for providing health care facilities within its province except in federally administered areas. In Pakistan, there are three tiers of governmental healthcare delivery systems, which comprise government, semi-government, and parastatal organizations. The purpose of this study is to understand the rapid increase in influx of patients in major city hospitals of the Punjab province. The reasons for patient flow towards the major city areas can vary, but our point of focus is mainly on those patients who are prescribed to get a computed tomography (CT) scan done for better diagnosis and early treatment, including but not limited to roadside accident cases. The study targets the define, measure, analyze, improve and control (DMAIC) problem solving approach to assess the potential cause of CT patient flow and the challenges that the health department is facing to cater to such a patient niche and process while minimizing the congestion in city government hospitals. The approach in this study summarizes with numerous quality tools like Voice of Customer (VOC) which is customer feedback, Customer Output Process Input and Supplier (COPIS) processes use for high-level process map to know what your customer value, Approver Resource Member Interested (ARMI) chart which is use to analyze the stakeholders on management of the project, evaluation with the help of fishbone diagram and house of quality, and for process improvement methodology we use the team brainstorming technique and the kaizen 5-why technique. The technique came up with an idea of a public-private partnership (PPP) project—partnership between public agency and private firm, as the health care industry in Punjab province is going through budgetary issues. In a PPP project, the Government will allocate the space to a private firm to build the facility on their own and provide quality service for CT scan diagnosis to the public of Punjab. The study identified the top 10 critical factors that the patients have expected from the government to be provided on priority. The Kaizen process improvement methodology has been adopted to provide the possible solution of government budgetary issues. The set of tools in this study can be adopted by other PPP projects to enhance the project performance.

## 1. Introduction

Every day, health care activities are increasing, making dependency on medical instruments and devices. The health industry, whether it is a small clinic or a teaching hospital, is in dire need of medical equipment, ranging from a simple surgical mask to a critical designed monitoring machine. In the medical industry, some devices are designed specially to give biochemical action that helps in better resolution of any damaged tissue or deeper clarification for any growing tumor [1]. To cure and assess the growing disease, the proper and effective diagnosis is the basic requirement. To have an accurate diagnosis, the team needs more sophisticated diagnosis and monitoring instruments. These monitoring machines will develop images of the body, and with the growth of computer science and imaging technology, medical science has become significantly dependent on imaging equipment [2]. Due to this dependency and accuracy in diagnosis, the Government of Punjab is experiencing the flow of patients in city government hospitals of the Punjab province, and a large number of patients are coming from districts where facilities for healthcare are not properly upgraded, especially in diagnostic equipment. The Punjab Growth Strategy (PGS) 2018 places an equal emphasis on accelerating economic growth and social outcomes, which include health.

The provincial government is currently focused on reforming the planning process to effectively operationalize PGS. Budgetary allocations in the Annual Development Plan are also being aligned to meet its health objectives, but they are still facing funding issues in this industry [3]. To tackle this budgetary issue, the Punjab government come up with the idea of a public-private partnership (PPP) which will also expedite the provision of health facilities at the selected 20 District Headquarter (DHQ) hospitals of Punjab, as a pilot project. The 36 districts in the province are provided with government hospitals, DHQs and Tehsil Headquarter hospitals (THQs), which are supposed to provide from free of cost (FOC) to bare minimum fees for the public; but due to budgetary issues and lack of equipment and trained and experienced staff, the people of the districts have to relocate to major city government hospitals for services in the treatment. Administratively, these hospitals are run by senior decorated doctors or medical superintendents who oversee a medical staff that comprises doctors, nurses, and other technicians; but due to the same reason as mentioned above, the quality of treatment is not reliable [4].

The main objective of the study is to address the unavailability of diagnosis services in Punjab districts, mainly focusing on the service of computed tomography (CT) scan facilities. The literature highlights the application and potential of the define, measure, analyze, improve and control (DMAIC) approach in the healthcare industry, and it highlights the importance and benefits of procuring the CT scanners without allocating a significant capital expenditure (CAPEX) via PPP, ultimately contributing to the customer (public) satisfaction in the health industry and the department of health. In addition, the DMAIC approach will also aid in upgrading the health facilities, potentially in areas that must be addressed on priority.

### 1.1. Research methodology

Six sigma (SS) is not only a quality management philosophy, but it is a comprehensive set of guidelines that enhances productivity, reduces variations and improves the overall quality of manufacturing and service processes [5]–[7]. SS was introduced by Motorola for its production accuracy, and later it become a buzz word for quality improvement. Many other industries, including health care sector, improved processes with six sigma improvement methodology [8]–[13]. The SS improvement process consists of five sequential steps known as DMAIC: define, measure, analyze, improve and control. DMAIC utilizes both statistical and managerial tools for process improvement and optimization [14]–[17]. Several authors discussed the application of DMAIC in the healthcare industry to improve the patient satisfaction in hospital service processes [18],[19].

This study focuses on the patients who are prescribed to have CT scan diagnosis done at DHQ level hospitals. The steps comprise the quality tools that help in the identification of possible contributing factors in the healthcare industry in Punjab province with the help of data gathering tools such as voice of the customer (VOC). The current process which is followed in the DHQ hospitals for CT scan patients is explained thoroughly via the customer output process input suppliers (COPIS) chart. Once the process is identified, the next step is to identify the stakeholders that will help to implement the new suggested process by using the ARMI chart (approver, resource, member, interested party) to define the roles and capacities of stakeholders. To identify and measure the levels of contribution of impacting factors, a cause and effect diagram is used, and then a weighted average table is drawn, taking responses from patients available at Punjab DHQ hospitals.

To analyze the potential of identified causes, a house of quality (HOQ) is built that also helps the PPP project for the identification of baselines to provide service quality against potential competitors. For further improvement in the system, the team brainstorming technique is utilized; and to control the process, the Kaizen 5-why technique is used, which also suggested further solutions in gray areas. The DMAIC approach is helpful for other service providers and also to improve the lean and JIT processes which may contribute to better service quality in the health industry [20].

## 2. Case study

Due to the modernization in disease diagnosis techniques, the healthcare industry is dependent on imaging technology. Because of this dependency and an exponential growth in population, the Government of Punjab (GoP) is experiencing rapid growth in patients in the district hospitals; and due to the unavailability of CT scan systems, these patients are referred to major city government hospitals. The patients travel along with their families and as a result create a bottleneck in clinical management at the major city hospitals.

This influx of masses is producing congestion in hospitals and also creating city traffic. The patients are from Punjab districts that are less modernized and less facilitated in imaging equipment, and due to this, the patient experiences lower quality services in treatment and ultimately relocates to the city hospitals of Punjab [21]. The study adopts the approach of the DMAIC tool to provide quality service in diagnosis using CT scan machines and also increase availability of the machines in DHQ hospitals so that people can avail themselves of scanning services in their own districts without traveling to far cities or spending higher costs on treatment in terms of traveling.

### 2.1. Define

In this phase, multiple factors are identified that are critical to quality from the customer's perspective. The study revolves around improving the quality of healthcare service and processes in the rural District Headquarter hospitals of Punjab [22]. The primary purpose of this phase is to point out the following:

1. The reason for public dissatisfaction with the departments of health in the regions.
2. The patient loads/clinical mismanagement in major city hospitals.
3. Unaffordability of CT scan systems by the Punjab government.
4. Status of vote banks in the upcoming elections.

With this, a public-private partnership has been proposed to provide CT scan facilities with minimum CAPEX. The study adopts the DMAIC approach to improve the process and identify and justify the actions that are critical to quality and critical to customers.

**Figure 1.**
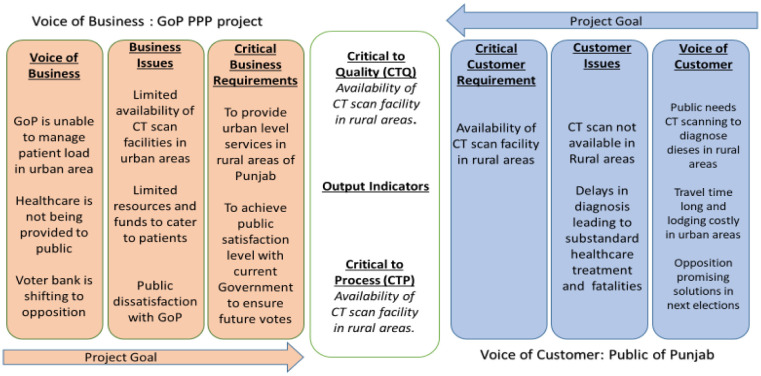
VOC vs. VOB.

#### 2.1.1. Voice of customer

In this process, VOC is gathered to know the first-hand knowledge of the situation, as the patients are immediate stakeholders in this project, and any input from patients on the research objective has added value to continue the work with concrete justifications. In Figure 1, the technique is employed to understand the customers' voices broadly. VOC is a technique that helps to identify the customers' desires from the project and also draft priorities according to their quality perspectives to serve them better in the project [23]. Figure 1 also indicates the interaction of the voice of business (VOB) with the voice of customers (VOC). The information is gathered from different district hospitals through informal surveys and observations. The result shows some output indicators which require consolidated solutions for both the business and customer and will serve as the CTQ parameter in the case study. The CTQ are as follows:

1. Availability of CT scaning at the DHQ hospitals.
2. Availability of CT technologists and supporting paramedical staff at the DHQ hospitals.
3. Availability of same-day reports at the DHQ hospitals.
4. Availability of CT scan engineers at the DHQ hospitals to assure minimum downtime.
5. Availability of CT scan backup power at the DHQ hospitals.

#### 2.1.2. COPIS

**Figure 2.**
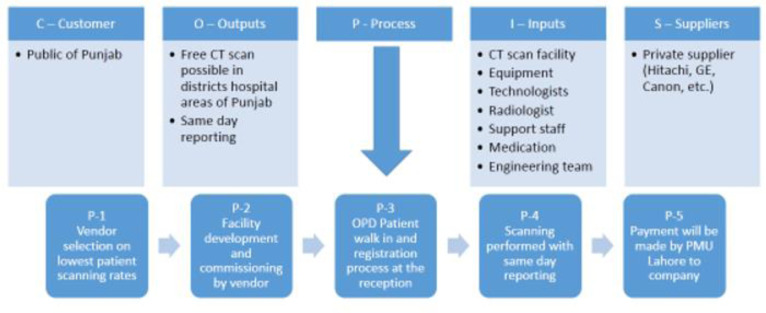
COPIS process map.

The next technique employed in the study is COPIS. The COPIS is also known as the SICOP process map, and the technique helps to draw a high-level process that needs a special responsibility to be addressed [24]. Figure 2 illustrates the flow of the process in the study. COPIS is used to identify the process, input, supplier, output and customer. Below are some pointers that were identified through the COPIS process chart. The department of health is responsible for allocating a location to private firms in each DHQ hospital to establish the facility for CT scans.

1. The supplier will provide the machinery and manpower to develop the site.
2. The supplier will develop the infrastructure and networking for robust data sharing.
3. The supplier will provide the patient's FOC registration, scan process, and reporting on the same day.
4. The supplier will provide the health department with patient data and scan data.
5. The DoH will pay per scan to the firm after a thorough audit by the PMU.
6. The customers (people of rural areas of Punjab) will have free scans and reports.
7. The customer will not have to travel to major cities for treatment and diagnosis purposes.
8. Better loads at major city hospitals may be managed.

#### 2.1.3. ARMI chart

The ARMI chart is used to communicate the roles and authorities of all the stakeholders of the project in the process of improvement. It also represents the level of involvement of each member at each phase of DMAIC [25]. Table 1 reflects the level of authority and responsibility of each stakeholder in the study.

It can be deduced from the table that the Punjab government is performing as a sponsor throughout the project life and has the capacity of approver at each phase in the project, except in the controlling phase. The private firms are acting as approvers and interested parties in different phases, while the public of Punjab, the customers of this project, holds the capacity of interested party.

**Table 1.** ARMI chart.

Stakeholder's role	Stakeholder resource	Define	Measure	Analyze	Improve	Control
Champion	Private firm	A	I	A	I	A
Sponsor	Government of Punjab	A	A	A	A	I
Mentor	Private firm	A	A	A	A	A
Process auditor	Project management unit-Lahore	A	I	I	I	A
Team member	Private firm-JV, DHQ hospital management	A	I	A	A	A
Project manager	Private firm	R/M	R/M	R/M	R/M	R/M
Subject matter expert (SME)	Private firm	R/M	R/M	R/M	R/M	R/M
Customer	Public of Punjab	I	I	I	I	I

### 2.2. Measure

The measure phase focuses on the measurement of factors that require action for improvement. The phase gives a model of variables and their current progress. The progress can then be compared and assessed easily and improved where required [26]. Below are some specially designed tools that support the measurement phase.

#### 2.2.1. Cause and effect diagram

**Figure 3.**
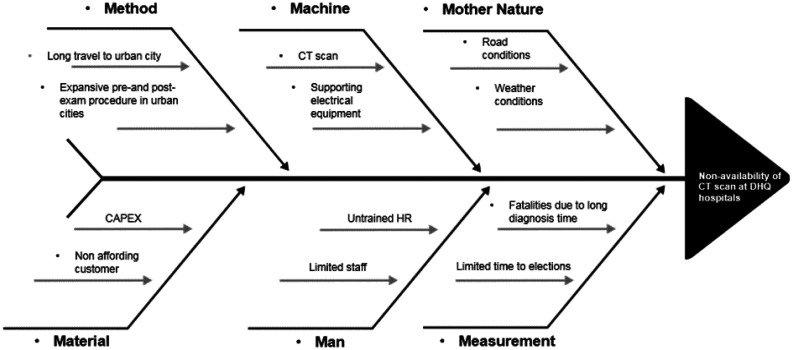
Ishikawa diagram to identify the causes of dissatisfaction in public of Punjab.

The cause-and-effect diagram is also known as the Ishikawa diagram. This tool helps the team to brainstorm different potential gaps. In this study, the core stakeholders of the project, including officials from GoP, the Health Secretary, the Health Department Team, the Project Management Unit, the Administrative Department of DHQs and the Project Team joined the brainstorming session to figure out different areas. Six categories are drawn in Figure 3 (manpower, machinery, measurement, material, method and Mother Nature) to identify the possible root causes of patient dissatisfaction [27].

### 2.3. Analyze

To analyze customer satisfaction in this study, the house of quality model has been used. The tool helps in the study to identify competitors of other regions and formulate a quality standard of scanning services for the local hospitals of Punjab districts. The study for the case will help in the load management of patients at major city hospitals [28].

**Figure 4.**
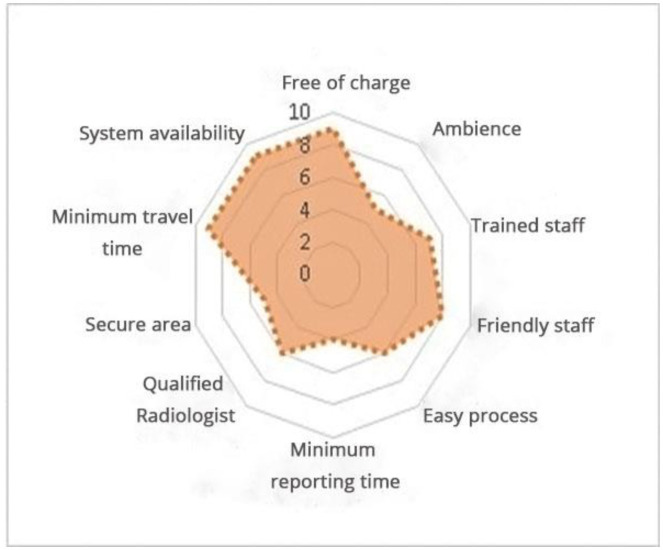
Voice of customer.

In the first step, the VOC (voice of the customer) has been classified based on the top 10 customer requirements (see Figure 4, data collected from the informal survey at DHQ hospitals). The data was collected through questionnaires and interviews. The patients were asked to identify the factors that they considered essential from the service provided. In this step, system availability, minimum travel time and free/subsidized service had the top weightage by the customers.

**Table 2.** Voice of customer.

Serial No.	Description	Weightage (out of 10)
1	Free of charge	9
2	Ambience	5
3	Trained staff	7
4	Friendly staff	8
5	Easy process	6
6	Minimum reporting time	4
7	Qualified radiologist	6
8	Secure area	5
9	Minimum travel time	9
10	System availability	9

**Figure 5.**
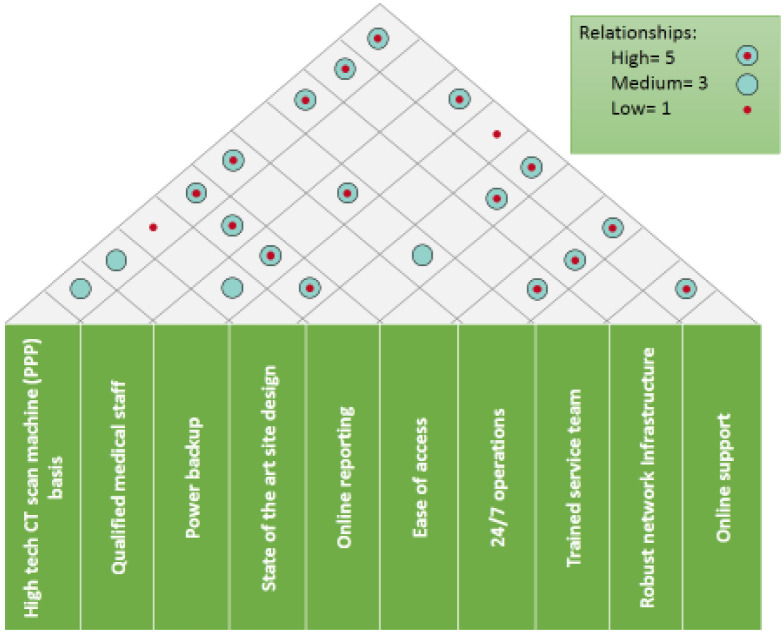
Technical attributes.

**Figure 6.**
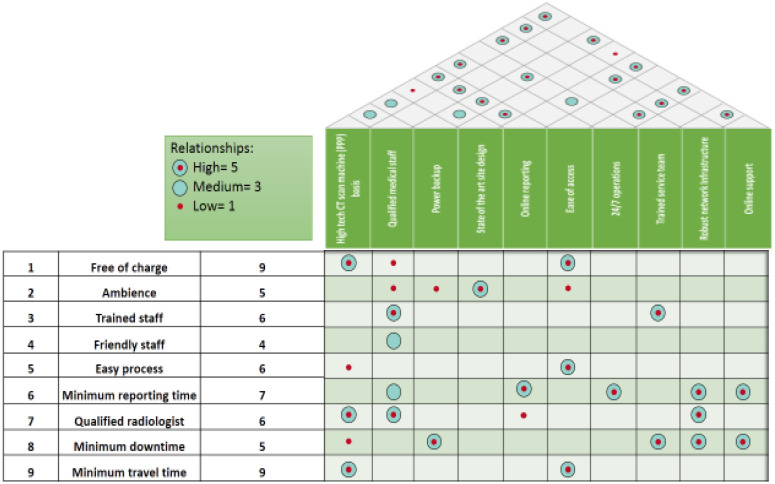
Inter-relation diagram.

**Figure 7.**
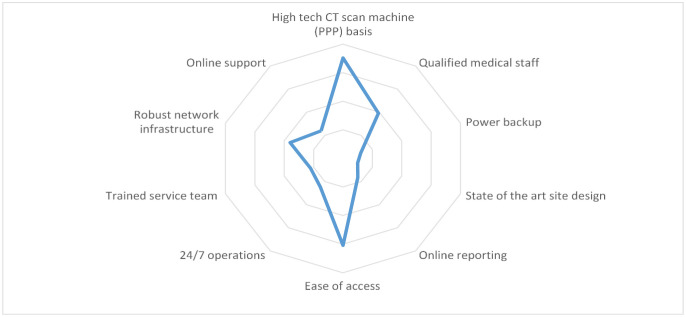
Inter-relations.

**Figure 8.**
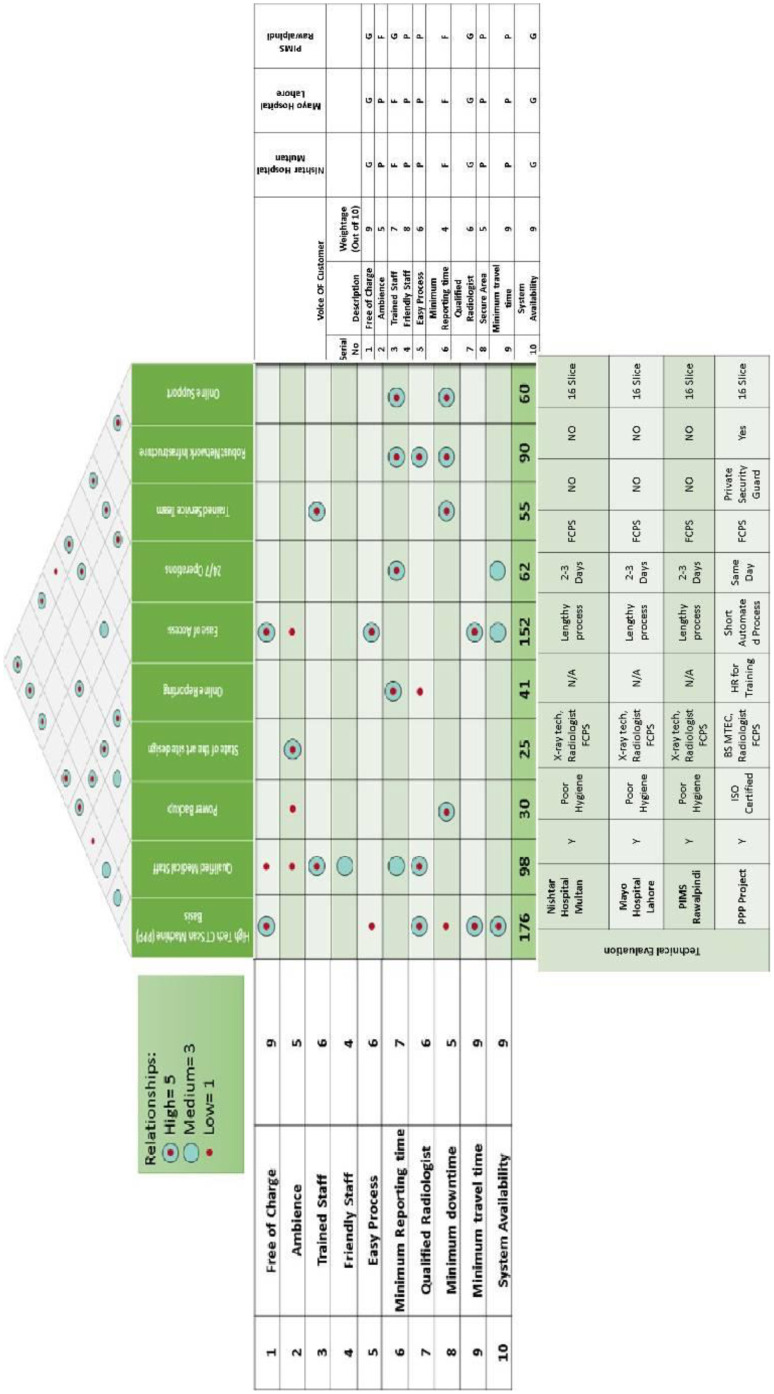
The house of quality.

In Figure 5, it is determined how to technically achieve the desired requirements and how these attributes are interrelated. Once it the internal interrelations were identified, correlations were generated between the technical attributes and voice of the customer, with applied weightage to identify the importance of each attribute (Figure 6). In the next step, as depicted in Table 3, currently available options who are competitors in this project are analyzed. At the final step, a comparison of the level of services between the proposed public-private partnership solution and potential competitors was created. This step shows and evaluates the services being offered and the future services to be offered by the solution against international standards (Table 4). The puzzle of the above steps is summarized in Figure 8 to generate the house of quality to better visualize the steps.

**Table 3.** Competitor assessment.

Voice of Customer			Nishtar Hospital Multan	Mayo Hospital Lahore	PIMS Rawalpindi
Serial No.	Description	Weightage (Out of 10)			
1	Free of charge	9	G	G	G
2	Ambience	5	P	P	F
3	Trained staff	7	F	F	G
4	Friendly staff	8	P	P	P
5	Easy process	6	P	P	P
6	Minimum reporting time	4	F	F	F
7	Qualified radiologist	6	G	G	G
8	Secure area	5	P	P	P
9	Minimum travel time	9	P	P	P
10	System availability	9	G	G	G

**Table 4.** Conformance to specifications.

Technical Attribute	Service Provider	Free of Charge	Ambience	Trained Staff	Friendly Staff	Easy Process	Minimum Reporting Time	Qualified Radiologist	Secure Area
Technical Evaluation	Nishtar Hospital Multan	Y	Poor hygiene	X-ray tech, radiologist FCPS	N/A	Lengthy process	2–3 days	FCPS	No
	Mayo Hospital Lahore	Y	Poor hygiene	X-ray tech, radiologist FCPS	N/A	Lengthy process	2–3 days	FCPS	No
	PIMS Rawalpindi	Y	Poor hygiene	X-ray tech, radiologist FCPS	N/A	Lengthy process	2–3 days	FCPS	No
	PPP Project	Y	ISO certified	BS MTEC, radiologist FCPS	HR for training	Short automated process	Same day	FCPS	Private security guard

### 2.4. Improve

In this phase, all the team members assemble to start work on the identified possible solutions. Implementation of the proposed solution is carried out with full efforts by every individual team member. The past work completed in the earlier phase complements improving the scanning facilities in DHQ hospitals and managing the patient load in city hospitals.

#### 2.4.1. 4-why 1-how approach

A kaizen root-finding technique was applied in the study to the possible range of solutions for the identified problems.

**Table 5.** Brainstorming meeting.

Step	1	2	3
*Topic*	Open Meeting	Kaizen Events	Closed Meeting
*Event*	A meeting is called to order to discuss the process	To find problem roots, 5 whys technique	Standardization of meeting
	Existing status of the survey for customer satisfaction	Implementing the kaizen project	Kaizen report data

The technique was carried forward by the PMU team in collaboration with other stakeholders. The team carried out the activity to formulate a response to reduce the patient load and address the budgetary problems. Table 6 elaborates the whole technique employed.

**Table 6.** Kaizen root finding technique.

X	WHY 1	WHY 2	WHY 3	WHY 4	Solution
Equipment Availability	Not available in all district hospitals	Poor planning	Incompetent planning staff		Private firm will provide equipment and staff without initial CAPEX
Staff Availability	Not available in all district hospitals	No equipment			PPP will provide equipment and staff without initial CAPEX
City	Location	Remote cities have long travel times	Transportation not always available	Road conditions are inconsistent	PPP will provide equipment at remote locations
Poor Patients	Non-affording patients				Scans will be free of cost to patient
Reporting Time	Can take up to week	Hospitals are in urban areas			Central reporting center will provide reports on same day via robust internet system
Shifts	Trauma system requires 24 hours operation	System not available at remote locations	Urban city hospital timings may not match travelling patients	Long travel times	Private firm will ensure 24 hours travel
Working Days	Trauma system requires 7 working days operation	System not available at remote locations	Urban city hospital timings may not match travelling patients	Long travel times	Private firm will ensure 7 days a week operation
Budget/Funds	Expensive machinery	Insufficient funds	PC1 not approved		Minimum CAPEX required to provide services in 20 districts

- **Equipment availability:** The most important problem that was identified was unavailability of equipment. So, making the equipment available will be the top priority for public satisfaction.
- **Staff availability:** Once the equipment is available, a team of qualified staff to run and maintain the equipment will be the next priority for public satisfaction.
- **City:** Most DHQ hospitals do not have CT scan facilities for their patients, which causes the people of districts to travel to major cities, causing bottlenecks in the city hospitals and public dissatisfaction.
- **Poor patients:** The general public at DHQ hospitals is not well to afford the service.
- **Reporting time:** CT scan is a trauma system, and it requires minimum reporting time in order to save lives.
- **Shifts and working days:** To establish well, the facility must operate 24/7 for patient satisfaction.
- **Budget:** Government has a great budget constraint, so a PPP is ideal to save CAPEX.

A priority chart has been generated to better understand the areas which require priority solutions. See Table 7.

**Table 7.** Priority chart.

Action	Ease (1)	Confidence (3)	Impact (5)	Total = I x C x E	Which process step is impacted in future state
Establish PPP with private firms to provide equipment without initial CAPEX	5 x 1 = 5	5 x 3 = 15	5 x 5 = 25	5 x 15 x 25 = 1875	P–1
PPP will provide equipment and staff without initial CAPEX	5 x 1 = 5	5 x 3 = 15	5 x 5 = 25	5 x 15 x 25 = 1875	
Firm will provide equipment to remote locations	5 x 1 = 5	5 x 3 = 15	5 x 5 = 25	5 x 15 x 25 = 1875	P–2, P–3, P–5
Scans will be free of cost to patient	4 x 1 = 4	3 x 3 = 9	5 x 5 = 25	4 x 9 x 25 = 900	
Central reporting center will provide reports on same day via robust internet system	3 x 1 = 3	3 x 3 = 9	5 x 5 = 25	3 x 9 x 25 = 675	P–4
Private firm will ensure 24 /7 Operation	3 x 1 = 3	2 x 3 = 6	5 x 5 = 25	3 x 6 x 25 = 450	
PPP will ensure minimum CAPEX required to provide services in 20 districts	2 x 1 = 2	5 x 3 = 15	3 x 5 = 15	2 x 15 x 15 = 450	P–1

**Table:** 

Key
Ease	1–5	5 = easiest
Impact	1–5	5 = highest
Cost	1–5	5 = lowest

### 2.5. Control

The last phase in the DMAIC approach is the controlling phase. In this phase, the study is finalized after generating a well communicated and strong list of strategies to control the new process suggested for implementation. In Table 8, the controlling plan is generated for significant process control.

**Table 8.** Controlling plan.

What is to be implemented	Where it is to be implemented	Who will implement it	By when	How it is to be implemented	Frequency of checking	Checked by
Terms of reference (TOR) will be published for invitation of EOI	Print and digital media	Health Department of Punjab	1 April 2017	Issuance of formal TORs by the technical committee	One time	Health Department Technical Committee
PPP will be established via tender on lowest patient rates	Print and digital media	Health Department of Punjab	1 May 2017	Tender award process by the technical committee	Process check	Health Department Technical Committee
CT scan machines will be installed phase wise (total 5 phases)	Phase wise selected District Headquarter hospitals	Private firm and Project Management Unit Lahore	1 June 2019	Import, logistics, installation and commissioning process will be carried out by private firm under the supervision of PMU	On each system delivery and monthly basis, whichever comes first	Private firm and Project Management Unit Lahore
Patient scan will be performed free of cost	All 20 District Headquarter hospitals	Private firm	1 June 2019	All operations of the site (patient registration, scanning, reporting) will be carried out by private firm	Monthly	Private firm and PMU
Reimbursement of claims made by private firm on scans performed	All 20 District Headquarter hospitals audited by PMU	Private firm and PMU	End of each month	Reimbursements will be made by the health dept. based on PMU audit	Monthly	Private firm and PMU
95% uptime of machines	All 20 District Headquarter hospitals	Private firm	Next 5 years	Competent and trained engineering team will ensure 95% uptime	Monthly	Private firm and PMU
Same day reporting	All 20 District Headquarter hospitals	Private firm	Next 5 years	Via robust internet system and team of qualified radiologists	Weekly	Private firm and PMU

## 3. Results

The above five-step process improvement methodology has been implemented in the project improvement process of customer satisfaction with diagnostic laboratories of the Punjab district in Pakistan. After successful implementation of all 20 CT scan systems in the selected district hospitals, an average of 10,000–12,000 patient influx on a monthly basis has been reduced. The diagnostic services are now available at the selected DHQ hospitals. When the Pandemic hit in 2020, this pilot project served as the primary diagnostic facility in the districts as HRCT (high-resolution CT) became curtailed in the early diagnosis of the coronavirus.

## 4. Conclusion and recommendations

The DMAIC methodology is an analytical approach to improving the public satisfaction level by implementing a process improvement technique. The DMAIC model put forward in this study provides a systematic approach for identifying, analyzing, improving and controlling the root causes of public dissatisfaction with the Government of Punjab. The tool allows the Department of Health in Punjab to identify the gaps in services of the patient niche and provide diagnostic equipment with a limited CAPEX. As the Punjab province is going through budgetary issues, it is concluded from the study that the Government can work on a public-private partnership (PPP) project, where the Government will allocate the space to a private firm to build the facility on their own and provide quality service for CT scan diagnosis to the public of Punjab.

The paper deals with the process of the DMAIC approach and its complementing tools to respond to the current situation of the healthcare industry in the Punjab district. The approach helps in achieving long-term objectives. Some recommendations must be carried forward to improve the situation.

1. The set of tools used in the study must be adopted by other hospitals in Punjab individually.
2. Evaluation and brainstorming sessions at the district level between maximum stakeholders should be arranged.
3. For in-depth and quality studies, more quality tools must be adopted by others. The healthcare industry is one of the most critical sectors of our society.
